# Effects of Fluorine-Based Modification on Triboelectric Properties of Cellulose

**DOI:** 10.3390/polym14173536

**Published:** 2022-08-28

**Authors:** Qiuxiao Zhu, Tingting Wang, Xiaoping Sun, Yuhe Wei, Sheng Zhang, Xuchong Wang, Lianxin Luo

**Affiliations:** Guangxi Key Laboratory of Clean Pulp and Papermaking and Pollution Control, School of Light Industrial and Food Engineering, Guangxi University, Nanning 530004, China

**Keywords:** cellulose, triboelectric nanogenerator, paper, contact electrification, wearable devices

## Abstract

The hydroxyl groups on the cellulose macromolecular chain cause the cellulose surface to have strong reactivity. In this study, 1H, 1H, 2H, 2H-perfluorodecyltriethoxysilane (PDOTES) was used to modify cellulose to improve its triboelectric properties, and a triboelectric nanogenerator (TENG) was assembled. The introduction of fluorine groups reduced the surface potential of cellulose and turned it into a negative phase, which enhanced the ability to capture electrons. The electrical properties increased by 30% compared with unmodified cellulose. According to the principles of TENGs, a self-powered human-wearable device was designed using PDOTES-paper, which could detect movements of the human body, such as walking and running, and facilitated a practical method for the preparation of efficient wearable sensors.

## 1. Introduction

With the rapid growth of global energy demand, the overexploitation of non-renewable fossil energy resources such as oil, coal, and natural gas has led to a serious energy crisis and environmental and ecological problems [1]. Therefore, how to obtain sustainable and environmentally friendly energy from the surrounding environment has attracted much attention. In 2012, Wang et al. proposed the concept of a triboelectric nanogenerator (TENG) [2,3], which is a technology based on the coupling effect of friction-generated electricity and electrostatic induction, with the advantages of low cost, small size, portability, and applicability to a variety of scenarios [4,5]. However, the relatively low output power density is still a limitation of the friction nanogenerator and is one of the main obstacles to the wide application of TENGs. Therefore, researchers have also made many attempts to enhance the electrical properties, such as the selection of suitable frictional electric materials, the introduction of functional groups [6,7], the doping of high dielectric materials [8], and the design of micropatterns [9,10]. The selection of suitable frictional materials is the most effective way to fundamentally improve the performance of TENGs.

Cellulose is the most abundant natural polymer compound on Earth, offering the advantages of low cost, good processability, good mechanical flexibility, and the ability to exhibit a unique combination of chemical, structural, dielectric, and optical properties [11,12,13]. These advantages and properties can make cellulose-based functional materials attractive as TENG substrates or components, a highly promising green material in the field of constituent materials for electronic devices. The polarity of frictional electrical materials is determined by the chemical properties of the material itself and is closely related to the functional groups present on the surface of the material [14]. The surface of cellulose is rich in hydroxyl groups, and by using hydroxyl groups in chemical reactions, some functional groups, chain segments, or molecules can be introduced to the cellulose molecules to synthesize cellulose derivatives. In recent years, many researchers have used chemical methods to modify various groups that promote electron transfer to cellulose to improve the frictional electrical properties. Yao et al. [15] reported a method to modify the frictional polarity of strong CNFs by chemically introducing nitro and methyl groups on the cellulose surface to enhance the frictional electrical output of TENGs, and after methylation and nitroxylation, the charge density of CNFs, respectively. Roy et al. [16] found that allicin from garlic juice enhanced the frictional electrical properties of cellulose nanofibers (CNFs) with strong efficacy, and the grafting of “thiol-ene” onto CNFs increased the TENG of pristine cellulose by about 6.5 times. Nie et al. [17] found that aminosilane modification of CNF films resulted in a significant enhancement of the positive charge on the CNF surface, allowing for excellent frictional charge density and hydrophobicity on CNFs. More studies have been conducted to modify cellulose in the positive direction, and fewer studies have been conducted to enhance the frictional negative polarity of cellulose. Fluorine is the most electronegative element with a small radius and a high ionization energy, and the introduction of the fluorine group is a reasonable choice to enhance the friction-negative polarity of cellulose.

In this study, 1H, 1H, 2H, 2H-perfluorodecyltriethoxysilane (PDOTES) was used to introduce fluorine groups with high electronegativity into cellulose, which improved the negative friction polarity of cellulose. FTIR, XRD, and XPS were used to characterize the modification of the cellulose, while SEM and AFM were used to examine the surface morphology and surface potential distribution of the modified cellulose. A TENG was assembled with modified cellulose as anode material and nylon (PA) film, and the triboelectric properties of this TENG were investigated. In addition, a TENG-based human wearable device is designed on this basis. At the same time, the prepared TENG can charge and power external devices, showing great potential in self-powered sensing systems and providing a factual basis for further expanding the application of friction materials.

## 2. Materials and Methods

### 2.1. Raw Materials and Reagents

Fast growing Eucalyptus cellulose fibers (Nanning, China). 1H, 1H, 2H, 2H-perfluorodecyltriethoxysilane (PDOTES) was purchased from Aladdin Biochemical Technology Co., Ltd. (Shanghai, China). A polymethyl methacrylate (PMMA) plate was purchased from Deyao Building Materials Co., Ltd. (Guangzhou, China), a conductive tape (copper) was purchased from Sitejie Office Store (Shenzhen, China), and a nylon (PA) film was purchased from Aofa Plastics Co., Ltd. (Suzhou, China).

### 2.2. Fluoro Modification of Cellulose

One gram of PDOTES was added to a 250 mL conical flask, 100 mL of ethanol/water solution at a volume ratio of 8:2 was added, and the mixture was stirred at room temperature for 2 h to obtain the hydrolysate of PDOTES. One gram of cellulose was added to the solution, and the mixture was stirred vigorously at room temperature for 5 min followed by sonication at 800 W for 10 min. After that, the solution was transferred to a 250 mL hydrothermal reactor and stirred in a constant temperature water bath at 80 °C for 6 h. After the reaction, the mixture was filtered and washed repeatedly with acetone and distilled water. The obtained product was PDOTES-cellulose.

### 2.3. Preparation of Paper and Fluorinated Paper (PDOTES-Paper)

**Figure 1:**
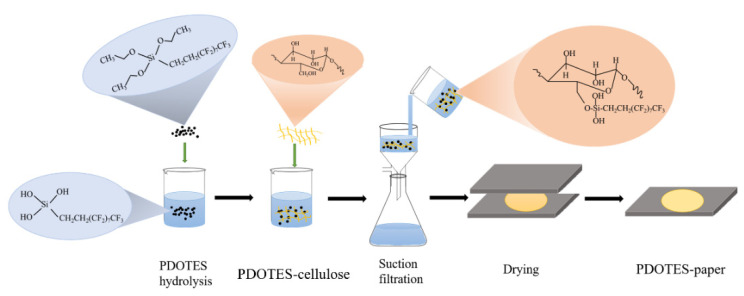
Preparation of PDOTES-paper.

The vacuum filtration method was employed to fabricate paper and PDOTES-paper films with a thickness of 50 μm. The paper-making process is shown in Figure 1. First, 0.6 g of cellulose or PDOTES-cellulose was placed in a 500 mL conical flask; then, 300 mL of distilled water was added. Next, the cellulose suspension was sonicated at room temperature for 10 min and then stirred for 2 h to disperse the cellulose evenly. A microporous filter membrane was placed at the bottom of a G5 sand core funnel, and the cellulose suspension was poured into it, forming a sheet of cellulose paper by vacuum suction filtration. After the sheet was formed, it was removed and placed in an automatic dryer. The temperature was set to 75 °C, and the drying time was set to 15 min. In this way, the paper and PDOTES-paper required for the experiments were obtained.

### 2.4. Structure of TENG and Wearable Device

**Figure 2:**
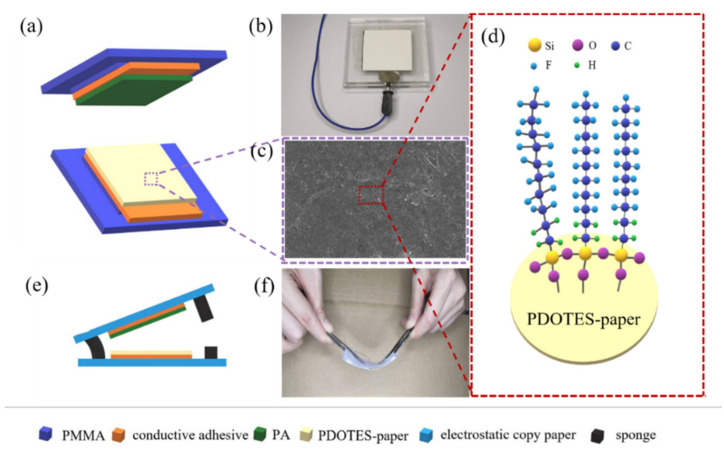
(**a**) Structural diagram of TENG; (**b**) TENG physical drawing; (**c**) SEM image of PDOTES-paper; (**d**) schematic diagram of TENG electrode; (**e**) diagram of wearable device structure; (**f**) physical drawing of wearable device.

The structures of the TENG and the wearable device are shown in Figure 2. An acrylic plate was cut into a 7 × 7 cm square as the TENG support material, and conductive double-sided tape, paper, PDOTES-paper, and PA film were cut into 4 × 4 cm squares. The conductive double-sided adhesive was attached to the middle of the acrylic plate, and then the paper, PDOTES-paper, and PA film were adhered to the conductive double-sided adhesive. The structure of the human-wearable device was similar to that of the TENG. It used xerographic paper as a soft support material, PDOTES-paper as a negative friction electrode material, and PA film as a positive electrode friction material, which were cut into 4 × 4 cm squares and fixed onto electrostatic copy paper with conductive double-sided tape. The two electrodes were connected with a sponge, and a layer of polyethylene film was coated on the outer layer of the wearable equipment to prevent erosion.

### 2.5. Characterization

After spraying gold on cellulose, scanning electron microscopy (SEM, Hitachi su8020, Tokyo, Japan) was used to observe the surface morphology and the elemental content, and atomic force microscopy (AFM, Dimension Edge, Bruker Co., Ltd., Berlin, Germany) was used to observe the surface roughness. KPFM images were taken by atomic force microscopy (AFM) at room temperature under dark conditions using an AFM system (XE-100, Park Systems, Bruker Co., Ltd., Berlin, Germany). The bias applied to the KPFM tip was 2.0 V for all samples. Changes in the functional groups of cellulose before and after fluoro modification were determined by Fourier transform infrared spectroscopy (FTIR, Vertes 70, Berlin, Germany) in the range 4000–400 cm^−1^. Changes in the surface elemental content of cellulose before and after fluorine modification were analyzed by X-ray photoelectron spectroscopy (XPS, Kratos Axis Ultra DLD, London, UK), and the change in crystallinity was analyzed by X-ray diffraction (XRD, Smartlab 3 kW, Tokyo, Japan). The crystallinity index (*CrI*) was calculated according to the Segal Formula (1):

(1) $$CrI\left(\%\right)=\frac{\left(I_{002}-I_{am}\right)}{I_{002}}\times 100\%$$

where *I_am_* is the intensity of the diffraction peak at 2θ = 18°, and *I*_002_ is the intensity of the diffraction peak at 2θ = 22°.

When testing the TENG, the periodic movement of the electrode was controlled by a tubular linear motor (Linmot H10–70 × 240/210, CA, USA) and a vibration exciter (JZK-10, Shenzhen, China), in which the tubular linear electrode controlled the motion frequency and the vibration exciter controlled the working pressure. The electrical signal generated by the TENG was collected using an electrometer (Keithley 6514, CA, USA) and output was sent to a Ni USB-6259, CA, USA, acquisition card.

## 3. Results

### 3.1. Characterization of Cellulose

#### 3.1.1. FTIR Analysis

**Figure 3:**
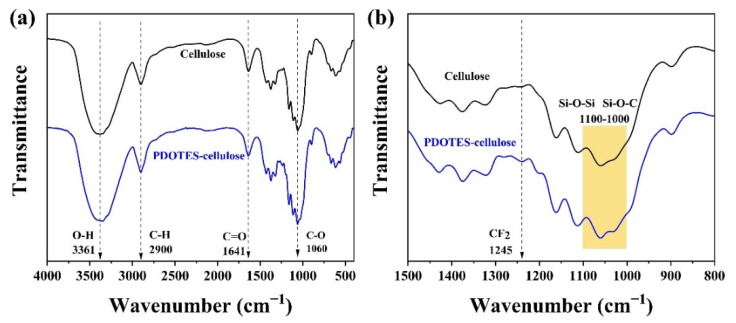
FTIR spectra of cellulose before and after modification: (**a**) 4000–500 cm^−1^; (**b**) 1500–800 cm^−1^.

FTIR can quantitatively and semi-quantitatively analyze samples. The increase in and loss of functional groups of cellulose before and after amino modification can be determined by FTIR reaction, and changes in peak strength can also indicate changes in functional group content [18]. As shown in Figure 3a, PDOTES-cellulose retains its absorption peak for cellulose. After the cellulose was treated with PDOTES, a new functional group absorption peak appeared in the infrared spectrum. As shown in Figure 3b, the absorption peak at 1245 cm^−^^1^ originates from the stretching vibration of -CF_2_ [19]. The absorption peaks at 1100–1000 cm^−1^ are attributed to Si-O-Si and Si-O-C [20]. These results preliminarily confirm that cellulose was successfully modified by PDOTES.

#### 3.1.2. XRD Analysis

**Figure 4:**
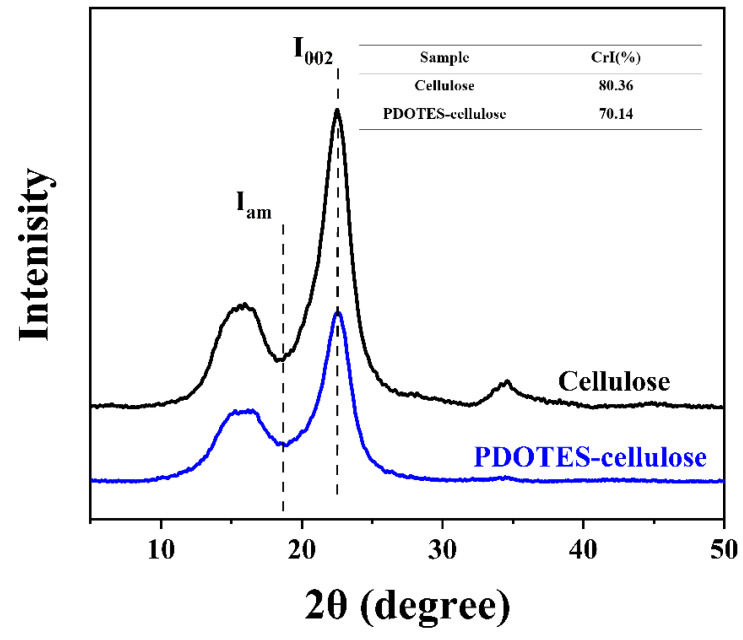
XRD spectrum of cellulose before and after modification.

XRD can be used to determine the structure and crystal morphology of cellulose and to measure changes in cellulose crystallinity [18]. As shown in Figure 4, the peaks at 2θ = 16° and 22° are diffraction absorption peaks of cellulose *I* [21], and indicate that fluorine-based modification did not destroy the crystalline region of cellulose, i.e., the cellulose was still a typical cellulose *I* after modification. It can be seen from Figure 4 that the diffraction peak intensity at *I*_002_ is significantly weakened after cellulose modification. After modification, the calculated crystallinity of cellulose decreased from 84.36% to 70.14%, owing to the introduction of amorphous PDOTES on the cellulose surface.

#### 3.1.3. XPS Analysis

**Figure 5:**
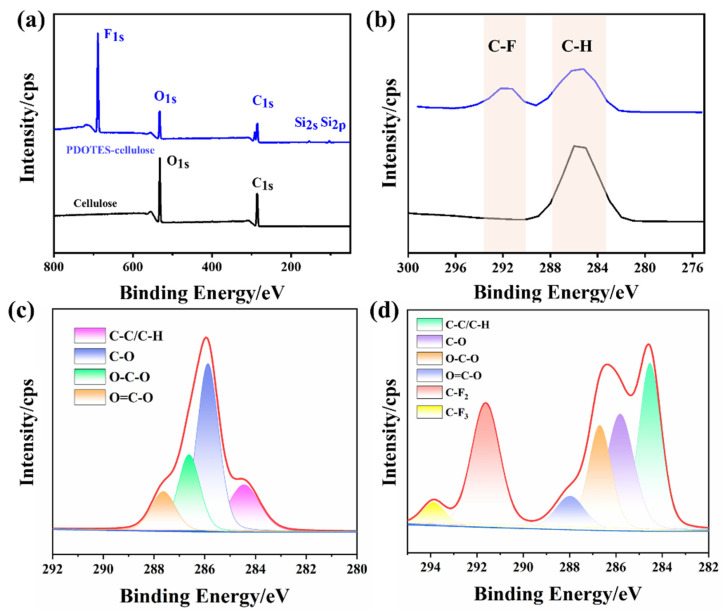
(**a**,**b**) Full XPS spectrum before and after cellulose modification; (**c**) C1s peaks on the surface of cellulose; (**d**) C1s peak deconvolution on PDOTES-cellulose surface.

XPS can be used for quantitative and semi-quantitative analysis of the surface elements of samples [22]. As shown in Figure 5a,b, peaks for both cellulose and PDOTES-cellulose appear at 532.4 and 286.5 eV [23], corresponding to O1s and C1s, respectively, and additional peaks for PDOTES-cellulose appear at 688.2, 153.4, and 102.2 eV, corresponding to F1s, Si2s, and Si2p, respectively. The proportions of elements on the surface of the sample analyzed by XPS are listed in Table 1. Compared with cellulose, F1s is present on the surface of PDOTES-cellulose, proving that the fluoro modification of cellulose was successful. From the XPS data, the F element is 37.64%, and the substitution degree of cellulose is calculated to be 0.77 [24]. At the same time, the C1s content of PDOTES-cellulose increased, while the O1s content and O/C decreased. This is because PDOTES was introduced into cellulose, and the amount of C1s on the cellulose surface increased significantly.

### 3.2. Surface Morphology and Elemental Distribution

**Figure 6:**
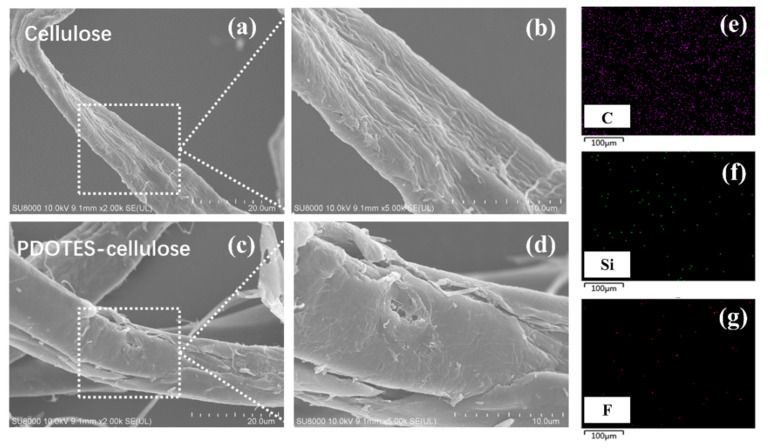
SEM images at different magnifications: (**a**,**b**) Cellulose; (**c**,**d**) PDOTES-cellulose; (**e**–**g**) surface elemental distribution of PDOTES-cellulose.

Figure 6a–d show SEM images of cellulose at different magnifications. Before modification, the surface of cellulose is smooth, the structure is dense, and there are no small particles on the surface. After modification, the surface of cellulose becomes rougher, holes and small particles appear, and fracturing of the cellulose can be observed. This shows that after modification, the crystalline area of cellulose was destroyed and glucoside bonds began to break, which is consistent with outcomes from XRD. Figure 6e–g depicts the surface element distribution of PDOTES-cellulose, where Si and F elements are present.

### 3.3. Surface Roughness and Surface Potential Distribution

**Figure 7:**
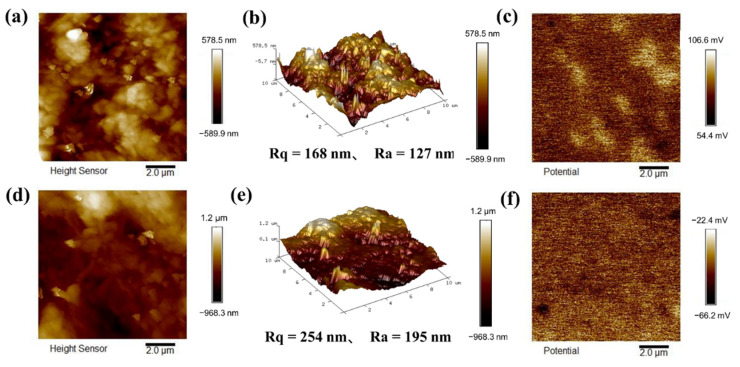
AFM images before and after modification: (**a**) 2D surface topography of paper; (**b**) 3D surface topography of paper; (**c**) surface potential of paper; (**d**) 2D surface topography of PDOTES-paper; (**e**) 3D surface topography of PDOTES-paper; (**f**) surface potential of PDOTES-paper.

Figure 7 shows that the surface roughness Ra of pure cellulose paper is 127 nm and that of PDOTES-paper is 195 nm. The surface roughness increases after modification, which corresponds to the situation of voids on the fiber surface after modification as observed by SEM. In order to determine the impact of chemical alteration on the material’s surface potential from a microscopic perspective and to investigate its triboelectric properties, the surface potential of cellulose was measured in the KPFM mode of atomic force microscopy (AFM). Figure 7c,f correspond to the surface potential of cellulose paper before and after modification. The surface potential of cellulose paper is 78.5 mV, and the surface potential of PDOTES-paper is −44.37 mV. The surface potential after fluorosilane modification not only decreased but also appeared in the opposite direction, which is due to the strong electronegativity of F, which made it have a strong electron-withdrawing ability and improved the negative frictional polarity of cellulose.

### 3.4. Working Principle of Triboelectric Nanogenerator

**Figure 8:**
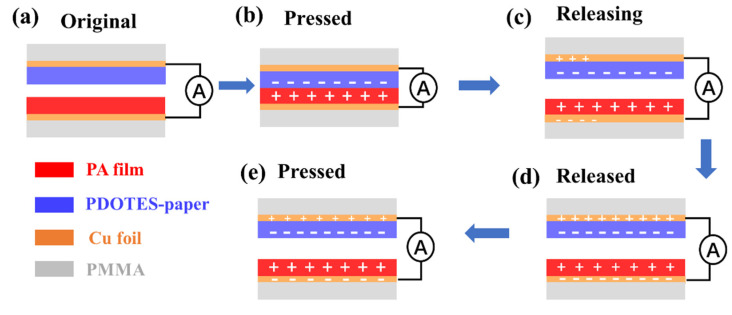
Structure and working principle of TENG. (**a**) PDOTES-paper-based TENG original structure; (**b**)TENG friction layers are in contact with each other by external force; (**c**) Remove the external force to separate the TENG friction layer; (**d**) The TENG friction layer is separated to the maximum distance; (**e**) Cyclic pair reapplying external forces.

Figure 8a–d show the working principle of a vertical contact separated TENG. In the initial state, as shown in Figure 8a, friction layers are separated from each other, and no charge is generated. As shown in Figure 8b, when the friction layers are pressed together, due to the different electron gain and loss abilities of the two friction layers, electrons from PA film are transferred to the surface of PDOTES-paper, so that the surface of PA film is positively charged, and the surface of PDOTES-paper is negatively charged. As shown in Figure 8c, when the pressure decreases and the electrodes are separated again, electrons flow from PDOTES-paper to PA film owing to the potential difference between the two friction layers. As shown in Figure 8d, when the friction layers stop separating, the circuit tends to balance, and no electrons move. As shown in Figure 8e, when the electrodes start to approach each other again, the potential of PDOTES-paper is higher than that of PA film, and the electrons move from PDOTES-paper to PA film. When the TENG electrodes carry out the above movements continuously, a stable alternating current is generated in the circuit [25].

### 3.5. Output Performance of Triboelectric Nanogenerator

**Figure 9:**
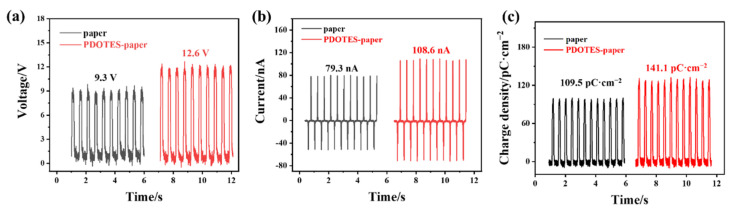
Output performance of paper-based TENG before and after cellulose modification: (**a**) Open circuit voltage; (**b**) short circuit current; (**c**) charge density.

Figure 9 shows the effect of fluoro modification on contact electrification of the cellulose. Using paper-based and PDOTES-paper-based TENGs as the experimental objects, a linear motor was used to move the two TENG friction layers through periodic contact separation movements. The maximum distance between the electrodes was 7 mm, and the acceleration was 0.5 m/s^2^. As shown in Figure 9, after fluorine-based modification of cellulose, the open circuit voltage of TENG increased from 9.3 to 12.6 V (a 35.48% increase), the short circuit current increased from 79.3 to 108.6 nA (a 36.71% increase), and the charge density increased from 109.5 to 141.1 pC·cm^−2^ (a 28.86% increase). This is because the fluorine group has a strong electronic function, which improves the negative polarity of cellulose and the output performance of the TENG, showing that the introduction of fluorine groups improves the contact electrification performance of cellulose.

**Figure 10:**
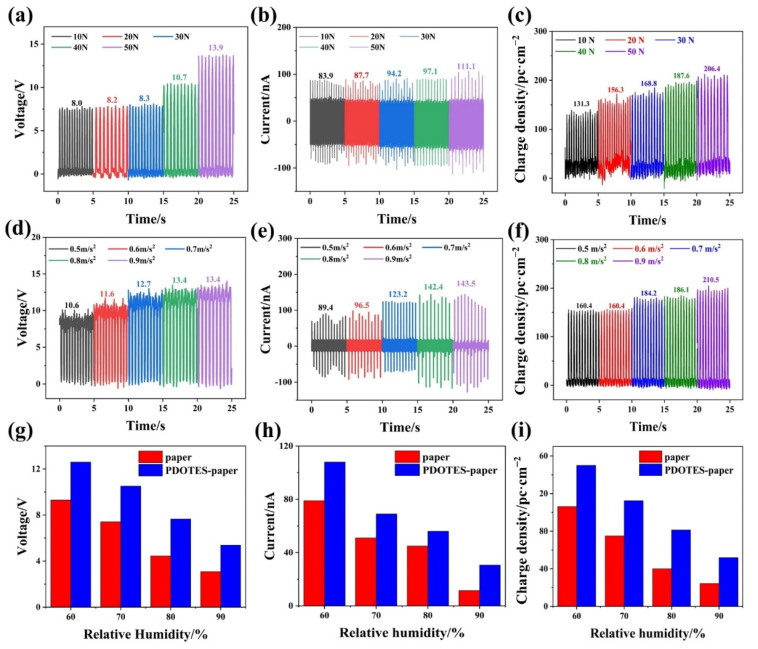
Effect of working conditions on PDOTES-paper TENG output performance. **(a**) Voltage, (**b**) current, and (**c**) charge density at various working pressures; (**d**) voltage, (**e**) current, and (**f**) charge density at various accelerations. The output performance of cellulose-based TENG before and after modification under different ambient humidity: Voltage (**g**), current (**h**), and charge density (**i**).

A change in working conditions greatly affects the output performance of the TENG. In the next experiment, a PDOTES-paper-based TENG was used as the experimental object to study the effect of working conditions on the output performance of the TENG. As shown in Figure 10a–c, when the contact pressure of the TENG was increased from 10 to 50 N using a vibration exciter, the open-circuit voltage, short-circuit current, and charge density were increased by 73.75%, 24.27%, and 57.20%, respectively. According to the analysis of the paper surface shown in Figure 7, the surface of the PDOTES-paper was rougher. With an increase in working pressure, PDOTES-paper gradually deforms and fills the gap with the PA film, so that the contact area between electrodes gradually increases, resulting in an improvement in the TENG output performance [26]. A linear motor was used to control the working acceleration of the TENG. As shown in Figure 10d–f, when the acceleration of indirect contact movement of the electrode controlled by the linear motor was increased from 0.5 m/s^2^ to 0.9 m/s^2^, the open-circuit voltage, short-circuit current, and charge density were increased by 26.42%, 60.51%, and 31.21%, respectively. Compared with the open-circuit voltage and charge density, the short-circuit current increased significantly more because the higher the acceleration of contact separation between electrodes, the shorter the time for electrons in the circuit to reach flow equilibrium, and the shorter the duration of the corresponding current peak, thus increasing the current peak.

In addition to the working pressure and working acceleration, the relative humidity of the environment is also an important factor affecting TENG output performance [27,28]. As shown in Figure 10g–i, the motion acceleration between the TENG electrodes was 0.5 m/s^2^, and as the air humidity in the control environment increased from 60% to 90%, the output performance of the paper-based and PDOTES-paper-based TENGs was reduced to varying degrees. The open-circuit voltage, short-circuit current, and charge density of the paper-based TENG decreased by 66.67%, 85.37%, and 77.05%, respectively, while the output performance loss of the PDOTES-paper-based TENG was less, with decreases of 57.14%, 71.72%, and 65.40%, respectively. This is because the introduction of a fluorine group into cellulose reduces the surface energy of PDOTES-paper and improves its negative friction polarity. On the other hand, taken in combination with the results in Figure 7, because the surface of PDOTES-paper is rougher than that of the paper, PDOTES-paper is less likely to be penetrated by water droplets in the air, so the PDOTES-paper-based TENG has stronger moisture resistance.

**Figure 11:**
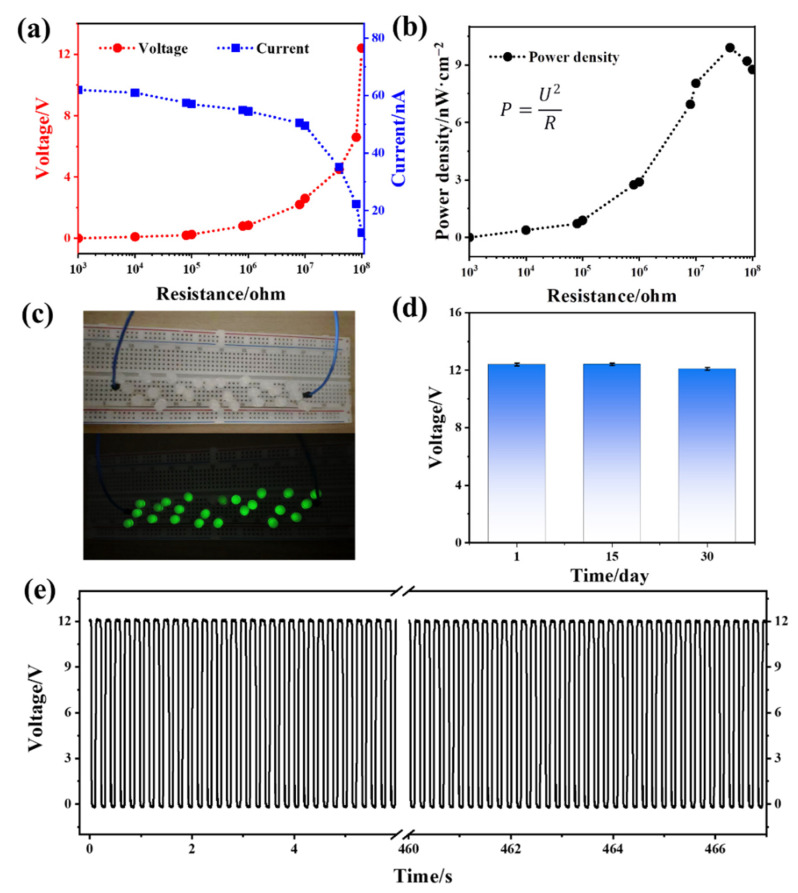
PDOTES-paper-based TENG: (**a**) Output voltage and current changes under external resistance; (**b**) power output curve under external resistance; (**c**) demonstrating supply of current to an LED lamp; (**d**) output performance vs. time; (**e**) output performance after 2000 cycles.

**Figure 12:**
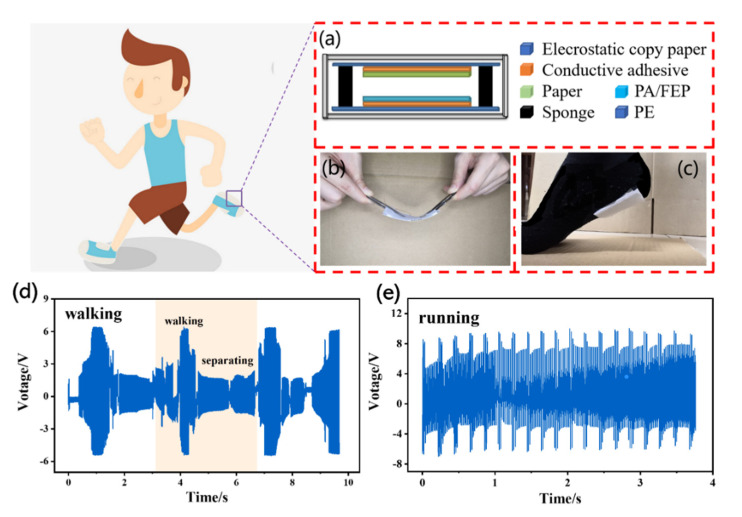
Structure diagram of the wearable device (**a**–**c**); open circuit voltage during walking (**d**); open circuit voltage during running (**e**).

TENGs can be used to supply power for small pieces of electronic equipment, so to study their output power we created a TENG-powered circuit [29,30]. As shown in Figure 11a,b, when the resistance of the variable resistance box in the TENG circuit increased from 10^3^ Ω to 10^8^ Ω, the output voltage of the TENG gradually increased, the output current gradually decreased, and the output power first increased and then decreased. When the resistance was 4 × 10^7^ Ω, the instantaneous power reached its maximum value of 9.9 nW·cm^−2^. When the PDOTES-paper-based TENG was connected to the LED circuit board, as shown in Figure 11c, more than 23 LED bulbs could be lit. The output stability of the TENG is an important index for evaluating its practical application [31]. As shown in Figure 12d,e, two stability tests were conducted on the PDOTES-paper-based TENG. First, the voltage was measured for 2000 continuous cycle operations, and was stable at approximately 12 V from the beginning to the end of the test. The other test was to measure the voltage of the TENG three times in one month. After 15 days, the voltage remained stable, and the voltage decreased slightly after one month.

### 3.6. Applications of FG-TENGs in Self-Powered Sensing

As a wearable device, a paper-based TENG is lightweight, thin, and flexible. It has potential for a wide number of applications as self-powered sensors [32,33]. As shown in Figure 12, a wearable device was manufactured with PDOTES-paper as a negative friction electrode adhered to socks. By simulating human movement, electrical signals were generated during the process of heel–ground contact separation. We performed two typical movements: walking and running. As shown in Figure 12, different electrical signals were detected. It can be seen from the electrical signal that the frequency of the electrical signal reflects the frequency of human movement. Moreover, when the movement state changes from walking to running, the peak value of the electrical signal also increases, because not only the working frequency of the wearable device increases, but the pressure exerted by the foot on the wearable device also increases. This shows that a paper-based TENG, as a self-powered sensor, can effectively monitor human motion.

## 4. Conclusions

The cellulose is chemically modified by 1H, 1H, 2H, 2H-perfluorodecyltriethoxysilane. The access of the fluorine group increases the surface roughness of cellulose, and the surface potential changes from a positive to a negative phase, so that the modified cellulose has negative triboelectricity. A TENG was prepared with PDOTES-paper as a friction-negative material. The output electrical performance was approximately 30% higher than that of a paper-based TENG. When the resistance in the working circuit was 4 × 10^7^ Ω, the output power reached 9.9 nW·cm^−2^. When connected to an LED circuit board, more than 20 LED bulbs could be lit at the same time. Using a PDOTES-paper-based TENG, a self-powered human-wearable device was designed to detect human movement. This shows that the contact electrification of cellulose has the potential for broad applications.

## Figures and Tables

**Table 1:** Cellulose surface element ratios.

Samples	C1s (%)	O1s (%)	F1s (%)	O/C (%)
Cellulose	55.72	44.28	-	79.47
PDOTES-cellulose	43.77	18.59	37.64	42.47

## Data Availability

Data available in a publicly accessible repository.

## Nomenclature

PDOTES	1H, 1H, 2H, 2H-perfluorodecyltriethoxysilane
TENG	Triboelectric nanogenerator
CNFs	Cellulose nanofibers
PMMA	Polymethyl methacrylate
PA	Nylon

## References

[1] Wei X., Zhao Z., Zhang C., Yuan W., Wu Z., Wang J., Wang Z.L. (2021). All-Weather Droplet-Based Triboelectric Nanogenerator for Wave Energy Harvesting. ACS Nano.

[2] Wang Z.L., Wu W. (2012). Nanotechnology-Enabled Energy Harvesting for Self-Powered Micro-/Nanosystems. Angew. Chem. Int. Ed..

[3] Niu S., Wang S., Lin L., Liu Y., Zhou Y.S., Hu Y., Wang Z.L. (2013). Theoretical study of contact-mode triboelectric nanogenerators as an effective power source. Energy Environ. Sci..

[4] Chen C., Chen L., Wu Z., Guo H., Yu W., Du Z., Wang Z.L. (2020). 3D double-faced interlock fabric triboelectric nanogenerator for bio-motion energy harvesting and as self-powered stretching and 3D tactile sensors. Mater. Today.

[5] Chandrasekhar A., Vivekananthan V., Khandelwal G., Kim S.J. (2019). A fully packed water-proof, humidity resistant triboelectric nanogenerator for transmitting Morse code. Nano Energy.

[6] Rajabi-Abhari A., Lee J., Tabassian R., Kim J., Lee H., Oh I. (2022). Antagonistically Functionalized Diatom Biosilica for Bio-Triboelectric Generators. Small.

[7] Yao L., Zhou Z., Zhang Z., Du X., Zhang Q.-L., Yang H. (2022). Dyeing-Inspired Sustainable and Low-Cost Modified Cellulose-Based TENG for Energy Harvesting and Sensing. ACS Sustain. Chem. Eng..

[8] Shi K., Zou H., Sun B., Jiang P., He J., Huang X. (2019). Dielectric Modulated Cellulose Paper/PDMS-Based Triboelectric Nanogenerators for Wireless Transmission and Electro polymerization Applications. Adv. Funct. Mater..

[9] Kim I., Roh H., Choi W., Kim D. (2021). Air-gap embedded triboelectric nanogenerator via surface modification of non-contact layer using sandpapers. Nanoscale.

[10] Mule A.R., Dudem B., Yu J.S. (2018). High-performance and cost-effective triboelectric nanogenerators by sandpaper-assisted micropatterned polytetrafluoroethylene. Energy.

[11] Zhao D., Zhu Y., Cheng W., Chen W., Wu Y., Yu H. (2021). Cellulose-Based Flexible Functional Materials for Emerging Intelligent Electronics. Adv. Mater..

[12] Zhang R., Dahlstrom C., Zou H., Jonzon J., Hummelgard M., Ortegren J., Blomquist N., Yang Y., Andersson H., Olsen M. (2020). Cellulose-Based Fully Green Triboelectric Nanogenerators with Output Power Density of 300 W m^−2^. Adv. Mater..

[13] Qin Y., Mo J., Liu Y., Zhang S., Wang J., Fu Q., Wang S., Nie S. (2022). Stretchable Triboelectric Self-Powered Sweat Sensor Fabricated from Self-Healing Nanocellulose Hydrogels. Adv. Funct. Mater..

[14] Wang N., Liu Y., Ye E., Li Z., Wang D. (2022). Control methods and applications of interface contact electrification of triboelectric nanogenerators: A review. Mater. Res. Lett..

[15] Yao C., Yin X., Yu Y., Cai Z., Wang X. (2017). Chemically Functionalized Natural Cellulose Materials for Effective Triboelectric Nanogenerator Development. Adv. Funct. Mater..

[16] Roy S., Ko H.-U., Maji P.K., Van Hai L., Kim J. (2019). Large amplification of triboelectric property by allicin to develop high performance cellulosic triboelectric nanogenerator. Chem. Eng. J..

[17] Nie S., Cai C., Lin X., Zhang C., Lu Y., Mo J., Wang S. (2020). Chemically Functionalized Cellulose Nanofibrils for Improving Triboelectric Charge Density of a Triboelectric Nanogenerator. ACS Sustain. Chem. Eng..

[18] Tabaght F.E., El Idrissi A., Aqil M., Elbachiri A., Tahani A., Maaroufi A. (2021). Grafting method of fluorinated compounds to cellulose and cellulose acetate: Characterization and biodegradation stund. Cellul. Chem. Technol..

[19] Nie S., Fu Q., Lin X., Zhang C., Lu Y., Wang S. (2021). Enhanced performance of a cellulose nanofibrils-based triboelectric nanogenerator by tuning the surface polarizability and hydrophobicity. Chem. Eng. J..

[20] Kaynak B., Alpan C., Kratzer M., Ganser C., Teichert C., Kern W. (2017). Anti-adhesive layers on stainless steel using thermally stable dipodal perfluoroalkyl silanes. Appl. Surf. Sci..

[21] French A.D. (2014). Idealized powder diffraction patterns for cellulose polymorphs. Cellulose.

[22] Xu S., Wu L., Lu S., Wu H., Huang L., Chen L. (2016). Preparation and Characterization of Cellulose-g-PFOEMA. J. Cellul. Sci. Technol..

[23] Jing-qiang Z., Lu L.I.N., Bei-hai H.E., Shi-jie L., Ping-kai O. (2009). X-Ray Photoelectron Spectroscopic Analysis of Celluloses with Different Crystallization Index. Chem. Ind. For. Prod..

[24] Levdanskya V.A., Kondracenkoa A.S., Levdanskya A.V., Kuznetsova B.N., Djakovitchc L., Pinelc C. (2014). Sulfation of Microcrystalline Cellulose with Sulfamic Acid in N, N-Dimethylformamide and Diglyme. J. Sib. Fed. Univ..

[25] Mi H.Y., Jing X., Zheng Q., Fang L., Huang H.-X., Turng L.-S., Gong S. (2018). High-performance flexible triboelectric nanogenerator based on porous aerogels and electrospun nanofibers for energy harvesting and sensitive self-powered sensing. Nano Energy.

[26] Xu Y., Min G., Gadegaard N., Dahiya R., Mulvihill D.M. (2020). A unified contact force-dependent model for triboelectric nanogenerators accounting for surface roughness. Nano Energy.

[27] Wen R., Guo J., Yu A., Zhai J., Wang Z.L. (2019). Humidity-Resistive Triboelectric Nanogenerator Fabricated Using Metal Organic Framework Composite. Adv. Funct. Mater..

[28] Wang N., Zheng Y., Feng Y., Zhou F., Wang D. (2020). Biofilm material based triboelectric nanogenerator with high output performance in 95% humidity environment. Nano Energy.

[29] Li Y., Cheng G., Lin Z.-H., Yang J., Lin L., Wang Z.L. (2015). Single-electrode-based rotationary triboelectric nanogenerator and its applications as self-powered contact area and eccentric angle sensors. Nano Energy.

[30] Mule A.R., Dudem B., Patnam H., Graham S.A., Yu J.S. (2019). Wearable Single-Electrode-Mode Triboelectric Nanogenerator via Conductive Polymer-Coated Textiles for Self-Power Electronics. ACS Sustain. Chem. Eng..

[31] Wu J., Xi Y., Shi Y. (2020). Toward wear-resistive, highly durable and high performance triboelectric nanogenerator through interface liquid lubrication. Nano Energy.

[32] He X., Zou H., Geng Z., Wang X., Ding W., Hu F., Zi Y., Xu C., Zhang S.L., Yu H. (2018). A Hierarchically Nanostructured Cellulose Fiber-Based Triboelectric Nanogenerator for Self-Powered Healthcare Products. Adv. Funct. Mater..

[33] Sriphan S., Charoonsuk T., Maluangnont T., Pakawanit P., Rojviriya C., Vittayakorn N. (2020). Multifunctional Nanomaterials Modification of Cellulose Paper for Efficient Triboelectric Nanogenerators. Adv. Mater. Technol..

